# In silico modeling of methadone-related ion current effects to predict QT interval prolongation

**DOI:** 10.1007/s10928-026-10059-2

**Published:** 2026-09-22

**Authors:** Jacob A. Lindsey, Omar A. Aboshady, Min Yue, Sara K. Quinney, James E. Tisdale, Brian R. Overholser

**Affiliations:** 1 Department of Pharmacy Practice, College of Pharmacy, Purdue University, West Lafayette, IN USA; 2 Clinical Pharmacology Department, Faculty of Medicine, Menoufia University, Menoufia, Egypt; 3 Department of Medicine, Division of Clinical Pharmacology, School of Medicine, Indiana University, Indianapolis, IN USA; 4 Department of Obstetrics and Gynecology, School of Medicine, Indianapolis, IN USA

**Keywords:** Methadone, QT interval, hERG, I_Kr_, I_K1_, Torsades de Pointes

## Abstract

Methadone is commonly associated with QT interval prolongation, which can lead to torsades de pointes. This effect has primarily been attributed to blockade of the delayed rectifier potassium current, I_Kr_. However, methadone inhibits other ion currents to varying degrees. A deeper understanding of how ion currents interact to produce drug-induced QT interval prolongation may support development of strategies to mitigate risk of potentially fatal arrhythmias. Given the difficulty of isolating the contributions of specific ion currents in drugs with multiple effects, this study used cardiac electrophysiology simulations to predict current-specific effects of methadone-induced QT interval prolongation. Action potential (AP) and electrocardiogram (ECG) models based on the Tusscher-Noble-Noble-Panfilov (2004) model were built using the Cardiac Safety Simulator version 2.2. Single-cell APs were simulated using published in vitro data of methadone’s effects (half-maximal inhibitory concentration and Hill coefficient) on multiple ion currents. The model was extrapolated to simulate ECGs using population parameters from a clinical study, and outputs from both models were compared to published data. A stepwise modeling approach isolated individual ion current contribution toward proarrhythmia. The final ECG model predicted heart rate-corrected QT intervals (QTc) within 1.5% of the observed data at four concentration ranges. Evaluation of individual ion currents toward QTc interval modulation suggested that I_Kr_ and I_K1_ have a similar impact on methadone-related QTc interval prolongation. This study highlights the potential contribution of multiple ion currents to methadone’s proarrhythmic effects and advances the understanding of ion current interplay underlying methadone-induced arrhythmia.

## Introduction

QT interval prolongation is closely linked to the risk of torsades de pointes (TdP). TdP is a polymorphic ventricular tachycardia associated with sudden cardiac death [1]. The QT interval, measured on an electrocardiogram (ECG) from the beginning of the QRS complex to the end of the T wave, reflects the time-dependent electrical activity generated by sequential action potentials (AP) in cardiomyocytes across different regions of the heart [2]. A heart-rate corrected QT interval (QTc) exceeding 500 milliseconds (ms) is linked to a two- to threefold increase in the risk of TdP, with each additional 10 ms of prolongation further elevating that risk by roughly 5–7% [3, 4].

The shape and duration of a cardiomyocyte AP is governed by the coordinated activity of ion currents, which mediate transmembrane voltage fluctuations. Alterations in ion current activity modify AP duration (APD), thereby influencing the length of the QT interval [5, 6]. Because the QT interval represents the duration of ventricular systole, it serves as a key indicator of ventricular repolarization [7]. As such, currents that influence repolarization are of critical interest in understanding the risk of QT interval prolongation. Historically, the rapid component of the delayed-rectifier potassium current (I_Kr_) has been the most commonly linked to drug-induced QT interval lengthening [8, 9].

While multiple physiological and pathological factors influence QT interval duration, drug-induced QT interval prolongation is a significant clinical concern stemming from disruption of the balance between depolarizing and repolarizing currents [10]. QT interval prolongation has historically been a leading cause of for drug candidates to be withdrawn from the market or fail during development stages [11–13]. To mitigate this risk, the United States (US) Food and Drug Administration (FDA) adopted recommendations for a thorough QT (TQT) study in 2005 that applied to most drug candidates seeking approval for US marketing [14]. Due to the rigid design and high cost of TQT studies, in silico modeling is gaining prominence for cardiac risk assessment, as reflected in the latest International Conference on Harmonisation (ICH) E14/S7B guidance which aims to support more informed decision-making and potentially reduce late-stage failures [15–18]. Additionally, the Comprehensive in vitro Pro-arrhythmia Assay (CiPA) initiative, advocates for a paradigm shift in torsadogenic risk assessment, emphasizing the integrated analysis of multiple cardiac ion currents rather than focusing solely on I_Kr_ [19]. The complexity of multi-channel interactions poses challenges for in vitro approaches [20]. In contrast, in silico modeling offers a controlled framework that uses mathematical representations of cardiac electrophysiology to evaluate the individual and combined effects of ion currents on cardiac function [21].

Methadone is a synthetic opioid analgesic drug commonly used in pain management and treatment of opioid use disorder that has been consistently associated with concentration-dependent QT interval prolongation [22]. Previous work by Mikkelson et al. [23] demonstrated that the Tusscher-Noble-Noble-Panfilov (2004) ventricular cardiomyocyte cell model (TNNP) [24] can be used to predict QT intervals in methadone patients by incorporating methadone-induced inhibition of I_Kr_, along with additional effects on the fast inward sodium current (I_Na_) and the L-type calcium current (I_CaL_) [23, 24]. However, Klein et al. [25] recently characterized methadone’s inhibition of the inwardly rectifying potassium current (I_K1_), which plays a key role in maintaining resting membrane potential stability and influences the shape of the action potential at late phases of repolarization [26]. Additionally, recent studies [27–30] have further characterized the inhibitory effects of methadone on I_CaL_, and I_Na_ [31].This study aims to determine whether inclusion of methadone-induced I_K1_ inhibition, alongside established I_Kr_ blockade, improves mechanistic and quantitative prediction of QTc prolongation by validating simulated QTc outcomes against clinical concentration–QTc interval relationships and isolating current-specific effects on APD_90_ and QTc.

## Materials and methods

### Cardiomyocyte model evaluation and selection

Simulations were performed using the numerical solvers implemented within the Cardiac Safety Simulator (CSS) version 2.2 (Certara, Sheffield, UK) to investigate the effects of racemic methadone on cardiac electrophysiology. The CSS platform integrates ion-current inhibition effects within validated models of human left ventricular electrophysiology to predict drug effects on both action potential and ECG readings [18]. Default time step, spatial resolution, and solver tolerances were used that have been optimized within the platform to ensure numerical stability and convergence across a range of electrophysiologic conditions. Stability of model outputs was confirmed by verifying consistent APD_90_ and QTc predictions across repeated simulations under identical conditions.

Three established human ventricular cardiomyocyte models available within the CSS were initially evaluated: O’Hara–Rudy, TNNP 2006, and TNNP 2004. All simulations were conducted using methadone concentration–time profiles and population parameters derived from Eap et al. [31]. Each model was parameterized using published IC₅₀ values for methadone across relevant cardiac ion channels. For the O’Hara–Rudy model, the late sodium current (I_NaL_) was included.

Model selection was based on the ability of candidate models to (1) reproduce clinically observed QTc–concentration relationships, including agreement in slope and mean QTc across concentration ranges; (2) capture experimentally observed trends in APD_90_ at therapeutically relevant methadone concentrations; (3) demonstrate physiologically plausible responses to multi-channel ion current inhibition; (4) maintain numerical stability and consistency across simulations; and (5) support extension from single-cell electrophysiology to tissue-level pseudo-ECG simulations. Preference was given to models with established use in drug-induced QT assessment and alignment with regulatory frameworks.

Among the evaluated models, the ten Tusscher 2004 model demonstrated the best agreement with observed QT intervals and was therefore selected as the base cardiomyocyte model for subsequent analyses. For all simulations, the TNNP model was used to mathematically represent cardiomyocyte electrophysiology [24].

### Literature search and evaluation

A literature search using PubMed was used to identify articles published between January 1960 and September 2025 that reported IC_50_ values (half-maximal inhibitory concentrations) and Hill coefficients (slope of the dose-response curve) describing the effects of R, S methadone on cardiac ion currents. The search included combinations of the keywords “methadone”, “ion current”, “IC_50_”, “inhibition”, “cardiomyocyte”, “QT prolongation”, “I_Kr_”, “calcium”, “calcium channel”, and “sodium channel”. Studies employing experimental conditions or assay platforms not directly comparable to the broader literature (e.g., standardized high-throughput electrophysiology systems with fixed protocols and temperature conditions) were excluded from the IC₅₀ analysis. Physiologic temperature (PT) values were prioritized for I_Kr_ because these data are consistently available from studies conducted under conditions aligned with regulatory expectations. For all other currents, the CSS does not provide equivalent temperature-specific parameterization options, and inputs were therefore selected from available literature.

Relevant studies reporting the effects of methadone on I_Kr_, I_K1_, I_Na_, and I_CaL_ were identified [25, 27, 32], and the extracted parameter values are summarized in Table 1. The arithmetic mean of reported IC₅₀ values was used as the model input. The IC_50_ for I_K1_ was derived from a single study and I_Kr_ values were highly consistent across studies (2.8-0.3.0 µM). Furthermore, for I_CaL_, alternative IC₅₀ representations including minimum reported values and log-transformed means were also extracted and considered during parameterization but did not meaningfully alter model outputs or the relative contributions of I_Kr_ and I_K1_ to QTc prolongation. Reported Hill coefficients (nH) were similarly fixed inputs for model parameterization and were not fitted to experimental data. Values were extracted from the literature and used as empirical descriptors of the concentration–response relationship. As nH did not vary substantially across studies, arithmetic averaging provided a representative input. Where coefficients were unavailable, a default value of nH = 1 was applied consistent with standard practice.

**Table 1:** Ion Current Effects for Model Input

IC_50_ and Hill coefficient^a^ (*n*_H_) used in model	IC_50_ from literature (µM)	Hill coefficient from literature	Reference
I _K1_ IC_50_: 1.5 n_H_: 0.7	1.5	0.7	Klein et al. 2022 [25]
I _Kr_^b^ IC_50_: 2.9 n_H_: 0.85	2.8	0.9	Tran et al. 2020 [27]
	3	0.86	Eap et al. 2007 [32]
	2.9	0.8	Klein et al. 2022 [25]
I _CaL_ (Ca_v_1.2) IC_50_: 10.03 n_H_: 0.875	5.5	1.1	Tran et al. 2020 [27]
	Phasic: 26.3 Tonic: 7.7	NA	Kuryshev. 2010 [29]^c^
	RT: 10.7, 4.6 PT: 5.4	RT 0.4,0.5 PT 1.4	Ren et al. 2022^d^ [28]
I _Na_ (Na_v_1.5) IC_50_: 36.7 n_H_: 1.23	Phasic: 11.2 Tonic: 5.5	NA	Kuryshev. et al. 2010 [29]^c^
	40.1	1.3	Tran et al. 2020 [27]
	90	1.15	Schulze et al. 2013 [30]

RT: Room Temperature; PT: Physiologic Temperature; NA: Not available

^a^The reported IC_50_ values and Hill coefficients in the left column are an average of identified values from the literature. ^b^I_Kr_ IC_50_ values were only included from studies that utilized HEK cells at physiologic temperatures. ^c^Phasic block was considered state-dependent block in this study, while the tonic IC_50_ was state-independent [29]. ^d^IC_50_ values for Ca_v_1.2 were captured under various conditions. These include the IC_50_ on the step current at room temperature (Ic_50_: 10.7, n_H_: 0.4), the ramp current at room temperature (IC_50_: 4.6, n_H_: 0.6), and the ramp current at physiologic temperature (IC_50_: 5.4, n_H_: 1.4) [28]

Klein et al. (2022) [25] was the reference study for validating the model performance in simulating single-cell cardiomyocyte action potentials. This study investigated the relationship between 3 methadone concentrations (0.1 µM, 1 µM, and 10 µM) and action potential duration at 90% repolarization (APD_90_) in isolated swine cardiomyocytes. Because 10 µM is supratherapeutic beyond clinical validation data, the two lower concentrations were considered for assessing model performance [33]. Despite known interspecies differences between swine and humans in electrophysiology, this study was considered the most appropriate benchmark for action potential comparison given the absence of analogous human data [34].

A clinical validation study was also identified to assess the model’s predictive capability on QT intervals derived from ECGs. Following the literature search, the following criteria were used to identify studies for clinical validation: (1) inclusion of chronic methadone users, (2) reporting of both plasma methadone concentrations and QTc intervals on an individual level, and (3) demographic characteristics closely matching those of Northern Europeans. The demographic parameter criterion was applied because the CSS evaluates cardiac electrophysiology based on Northern European physiology [23], and differences in ethnicity may affect the comparison of cardiac responses to methadone. Eap et al. [32] was selected as the primary reference study due to its demographic alignment with the CSS. The study evaluated 179 chronic methadone users and identified a positive correlation between R, S methadone plasma concentration and QTc interval duration. Similar associations between methadone dose or plasma concentration and QTc interval prolongation have been documented across different study populations [35–38], further supporting the reliability of Eap et al. as a reference dataset.

For purposes of comparison between the observed and simulated studies, subject-level data on QTc interval, APD_90_ duration, and methadone concentration were extracted from the published figures using a web plot digitizer (WebPlotDigitizer, version 5.2, Automeris) [39].

### Action potential modeling approach

To replicate the conditions of the reference study, action potentials were simulated in individual midmyocardial cardiomyocytes at methadone concentrations of 0.1 µM, 1 µM, and 10 µM, incorporating ion current effects on I_Kr_, I_K1_, I_CaL_, and I_Na_. The relative inhibition of ion current conduction was determined within the CSS model according to the following equation:

$$\:Inhibition\:factor=\:\frac{1}{1+{\left(\frac{{IC}_{50}}{Concentration\left(\mu M\right)}\right)}^{{n}_{H}}}$$

where IC_50_ represents the half-maximal inhibitory concentration and *n*_*H*_ is the Hill coefficient. The TNNP model calculates the total transmembrane ionic current (I_ion_) as the summation of individual ion current components, represented by:

$$\begin{array}{c}\:I_{ion}=\:I_{Kr}+I_{K1}+I_{CaL}+I_{Na}+I_{to}+I_{Ks}\\+I_{NaCa}+I_{NaK}+I_{pCa}+I_{pK}+I_{bCa}+I_{bNa}\end{array}$$

For the purpose of the simulations in this study, I_Kr_, I_K1_, I_CaL_, and I_Na_ were manipulated to reflect methadone-induced inhibition, while all other currents were maintained in their uninhibited states.

### Physiological variability and action potential simulations

Action potentials were simulated in 20 virtual midmyocardial cells at each methadone concentration used by Klein et al. (2022) [25]. While different cardiac cell types would have influenced the results, midmyocardial cells represent the longest baseline APD, the highest sensitivity to repolarization, and are the primary drivers of the QT interval. Furthermore, these were important to use to replicate the in vitro work by Klein et al. (2022) [25].

Physiologic variability based on age and sex were incorporated, including differences in cardiomyocyte volume, sarcoplasmic reticulum volume, cardiomyocyte area, and ionic concentrations of potassium, sodium, and calcium. For both males and females, cardiomyocyte volume was modeled using a log-normal distribution with parameters β1 = 7.36346, β2 = 0.04551, and σ = 0.29602. Sarcoplasmic reticulum volume was fixed at 6% of cardiomyocyte volume. Cardiomyocyte area was modeled with β1 = 0.86, β2 = 0.002939, and σ = 0.102.

Sex-specific physiological variability in extracellular ionic concentrations was incorporated using the default Cardiac Safety Simulator (CSS) v2.2 population model. Mean (coefficient of variation [CV]) potassium concentrations were 4.21 mM (8.24%) for males and 4.09 mM (10.89%) for females. Mean sodium concentrations were 140.1 mM (2.15%) in males and 138.2 mM (3.18%) in females, while mean calcium concentrations were 2.42 mM (8.23%) and 2.31 mM (5.87%) for males and females, respectively.

The age range varied between 18 and 65 years, and 50% of the population was female for the 20 virtual cells generated per concentration. Total simulation time was set to 10,000 ms for all runs. The final simulated APD_90_ values were averaged across the 20 cells for each concentration and compared to the percent change in APD_90_ observed in the Klein et al. study [25]. Predicted (simulated) percent change was calculated relative to a simulation without ion current inhibition in the same virtual cardiomyocytes.

### Pseudo-ECG modeling approach

The AP model was extended to simulate tissue-level cardiac electrophysiology. To replicate the characteristics of the ECG reference study (Eap et al. [32]), a virtual population was generated using the CSS and Simcyp Simulator (version 21; Certara, Sheffield, UK). Demographic parameters, including sex, age, and ethnicity, along with individual methadone concentrations were extracted from the reference study to inform the virtual population. Due to the absence of patient-level data, three virtual patients were simulated per observed methadone concentration to reduce variability in cardiac physiology and facilitate comparison between the simulated and observed populations.

The aforementioned AP model parameters were applied to a one-dimensional string of cardiomyocytes representing ventricular tissue. The TNNP model was used to generate a pseudo-ECG for each virtual patient. Because the model only includes representations of ventricular cells, the simulated ECGs do not reflect atrial activity, resulting in the absence of P waves in the simulated ECGs.

To capture the structural differences in ventricular wall thickness that influence electrical conductance, the String Length Sjogren model [40] was incorporated. This model accounts for sex- and age-related variability in left ventricular wall thickness. Furthermore, the TNNP model captures differences in ion currents across the endocardial, midmyocardial, and epicardial layers of the heart. This transmural heterogeneity was incorporated in the pseudo‑ECG simulations with the distribution of cell types as 50% endocardial, 30% midmyocardial, and 20% epicardial, with a mean diffusion coefficient of 0.0016 cm^2^/ms. Fiber length was allowed to vary according to the physiological ventricular wall thickness model implemented in CSS. Consequently, fiber length was not fixed but reflected the simulated ventricular wall thickness for each virtual subject. The simulations employed the default Forward Euler integration scheme implemented within CSS v2.2 with a simulation time step of 1 ms. Absolute and relative solver tolerances were both set to 1 × 10⁻⁸.

Simulated QT intervals were corrected for heart rate using Bazett’s formula [41] to align with the methodology of the reference study:

$$\:QTc=\:\frac{QT}{\sqrt{RR}}$$

Model performance was evaluated using linear regression and goodness-of-fit plots. To compare QTc trends between predicted and observed data, linear regression models were fit to both datasets. Additionally, mean QTc interval values were compared quantitatively across four methadone concentration ranges (0-0.75, 0.75–1.25, 1.25-2, 2-5.15 µM) that each represented approximately 25% of the data. ΔQTc trends were also analyzed to account for baseline QT interval variability. ΔQTc intervals from both groups were grouped into 25 bins and averaged within each bin. Predicted ΔQTc intervals were calculated as the difference in QTc interval for the same virtual individuals between the full methadone simulation and a simulation without ion current inhibition. Since baseline QTc intervals were not available in the reference study, observed ΔQTc values were calculated as the observed QTc interval minus the mean QTc interval from the no-inhibition simulation.

### Mechanism based assessment

After validation of the full models, individual ion current contribution towards methadone-related QTc and APD_90_ interval prolongation was evaluated. This was accomplished through a stepwise simulation approach, where inhibition was applied to one ion current at a time while holding the others constant. For the ECG model, the effect of each isolated current block was quantified by calculating the ΔQTc interval relative to the no-inhibition simulation.

### Statistical analysis

Statistical analysis was performed using R (version 4.4.2) [42] and Rstudio (version 2024.12.0) [43]. The packages ggplot [44] and RColorBrewer [45] were used for data visualization. Comparisons of reported means for APD_90_ percent change versus simulated values were performed using Student’s unpaired t-test. For the ECG model, differences among the observed data, the full-model simulation, and the individual ion current inhibition simulations were assessed by two-way ANOVA followed by Tukey’s Honestly Significant Difference (HSD) post hoc test. A two-sided P value < 0.05 was considered statistically significant for all analyses.

## Results

### Effects of methadone on cardiac APD_90_

**Fig. 1:**
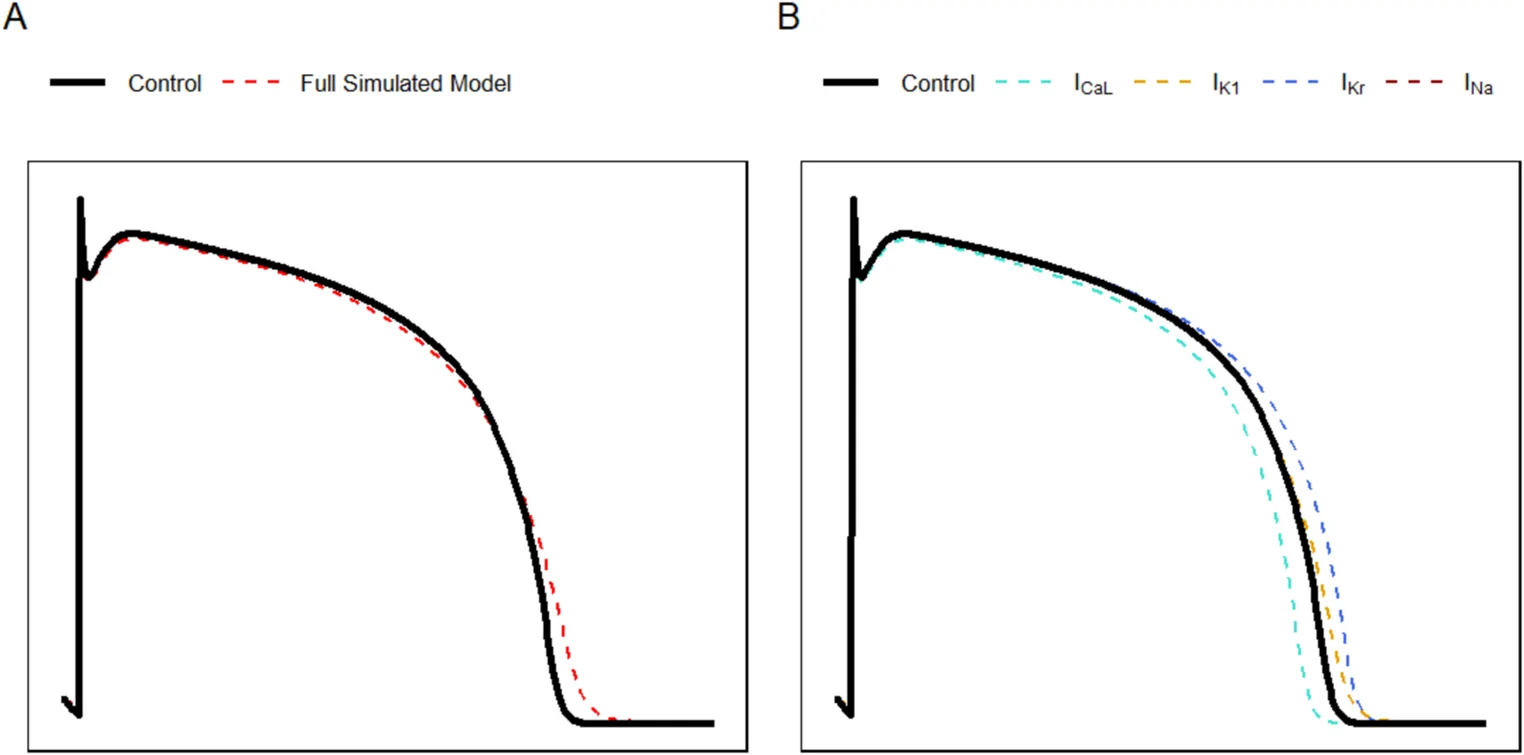
Methadone effect on APD_90_ in a randomly selected virtual cardiomyocyte in the 1 µM exposure group. (**a**) the black trace represents the baseline AP in the absence of methadone, while the dashed red trace shows the same AP following exposure to 1 µM methadone (**b**) Individual ion current contributions to APD_90_ prolongation are shown with each ion current (I_Kr_, I_K1_, I_Na_, and I_CaL_) inhibited individually at methadone a concentration of 1.0 µM (29%, 43%, 1.0%, and 11% blockade, respectively). The AP under selective block of I_Na_ is indistinguishable from the baseline AP

All AP simulations consisting of 20 left ventricular cardiomyocytes demonstrated that methadone prolongs APD_90_ in a concentration-dependent manner. At 1 µM, the model-predicted APD_90_ increased from a baseline of 356 ms to 372 ms (Fig. 1A). Figure 1B illustrates the individual contributions of ion current inhibition to APD_90_ prolongation.

Inhibition of I_Kr_ and I_K1_ produced comparable increases in APD_90_ (19 ms and 12 ms, respectively), while I_CaL_ inhibition shortened APD_90_ by 17 ms. I_Na_ inhibition had negligible impact, with an APD_90_ of 356 ms, which closely approximated the baseline value.

Predicted values were compared to observed cellular APs reported in the reference study by Klein et al. [25], which investigated APD_90_ changes in swine ventricular cardiomyocytes. The model captured experimental trends at both of the therapeutic concentrations (*p* > 0.05), as shown in Table 2. In the reference study, experimental APD_90_ increased up to 1 µM but decreased below baseline at 10 µM (-3.6% change from baseline). This supratherapeutic effect was not observed in the simulations (*p* < 0.05), which instead predicted progressive action potential prolongation (+ 12.4% change from baseline).

**Table 2:** Comparison of APD_90_ Percent Change for Simulated and Observed Data

Concentration	APD_90_ Percent Change ± SEM	
	Predicted^a^	Klein et al. 2022 [25]
0.1 µM	1.1 ± 2.6	4.7 **±** 1.6
1 µM	4.8 ± 2.6	6.5 **±** 1.7
10 µM*	12.4 ± 3.2	-3.6 **±** 3.6

*p < 0.05; SEM: Standard Error of the mean

^a^Percent change and standard errors were calculated relative to the mean simulated APD_90_ at 0 µM methadone (baseline condition)

### ECG model virtual population

The virtual population generated with Simcyp closely resembled the study population parameters described in Eap et al. [32], as detailed in Table 3.

**Table 3:** Demographics Profile of Simulated and Observed Patient Populations

Parameter	Simulated	Observed
Age in years (range)	21–52	21–53
Sex (% female)	21%	22%
Race (%)	100% White	96% White/ 4% Other

Due to the influence of heart rate on QTc interval measurement, heart rate was assessed to ensure validity. The average heart rate for the virtual population was 72 bpm, consistent with mean values ranging from 69 to 71 bpm in chronic methadone users [35, 38, 46, 47].

### Effects on QTc interval

The pseudo-ECG simulation incorporating all methadone-related ion current inhibition reproduced observed QTc interval trends with deviations of less than 1.5% across all tested concentration ranges (Table 4**)**.

**Table 4:** Comparison of Observed and Predicted QTc Intervals

	Observed Data	Simulated Data	Comparison
Methadone concentrations (µM)	Mean QTc ± SEM (ms)	Mean QTc ± SEM (ms)	Simulated/Observed Ratio
0–0.75 µM	413 **±** 24.2	407 **±** 27.3	0.985
0.75–1.25 µM	416 **±** 23.8	421 **±** 36.7	1.012
1.25–2 µM	422 **±** 22.3	425 **±** 35.0	1.007
2–5.15 µM	437 **±** 25.5	432 **±** 36.9	0.989

**Fig. 2:**
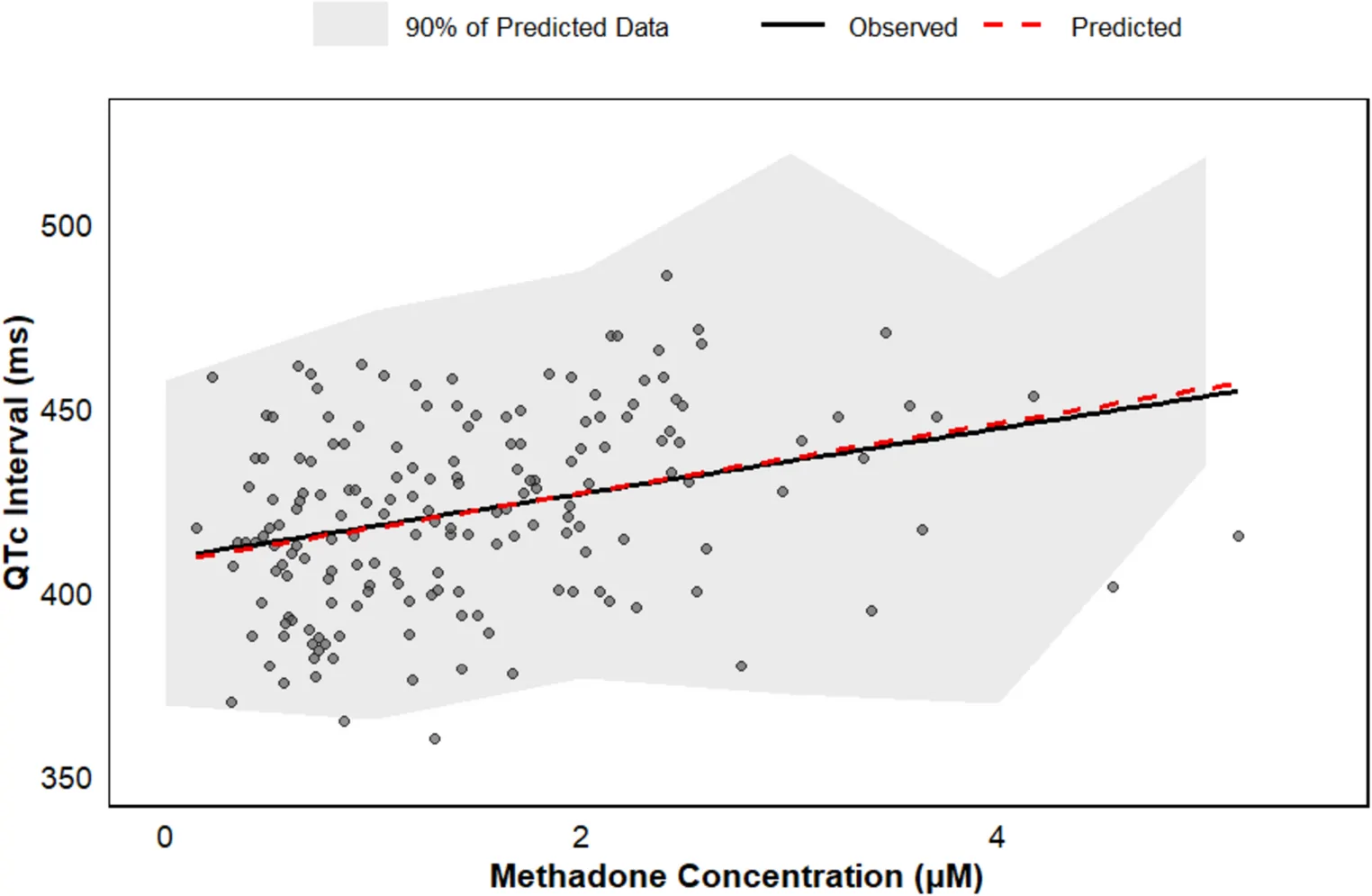
Comparison of predicted and observed QTc interval data. A linear regression model of the predicted data (red line) is overlaid with the regression model of the observed clinical data (black line). Individual data points represent observed QTc intervals at corresponding methadone concentrations. The shaded grey area denotes 90% of the simulated data. The regression equation for the observed data was $\:QTc=409.06\:+\:8.83\left(C\right)$, and for the simulated data, $\:QTc=407.57\:+\:9.28\left(C\right)$, where C represents methadone concentration in µM

No statistically significant differences in QTc intervals were detected between the observed and simulated groups (*p* > 0.05). Figure 2 illustrates the linear regression fits for both the simulated (r^2^ = 0.056, *p* < 0.001) and observed (r^2^ = 0.097, *p* < 0.001) data.

**Fig. 3:**
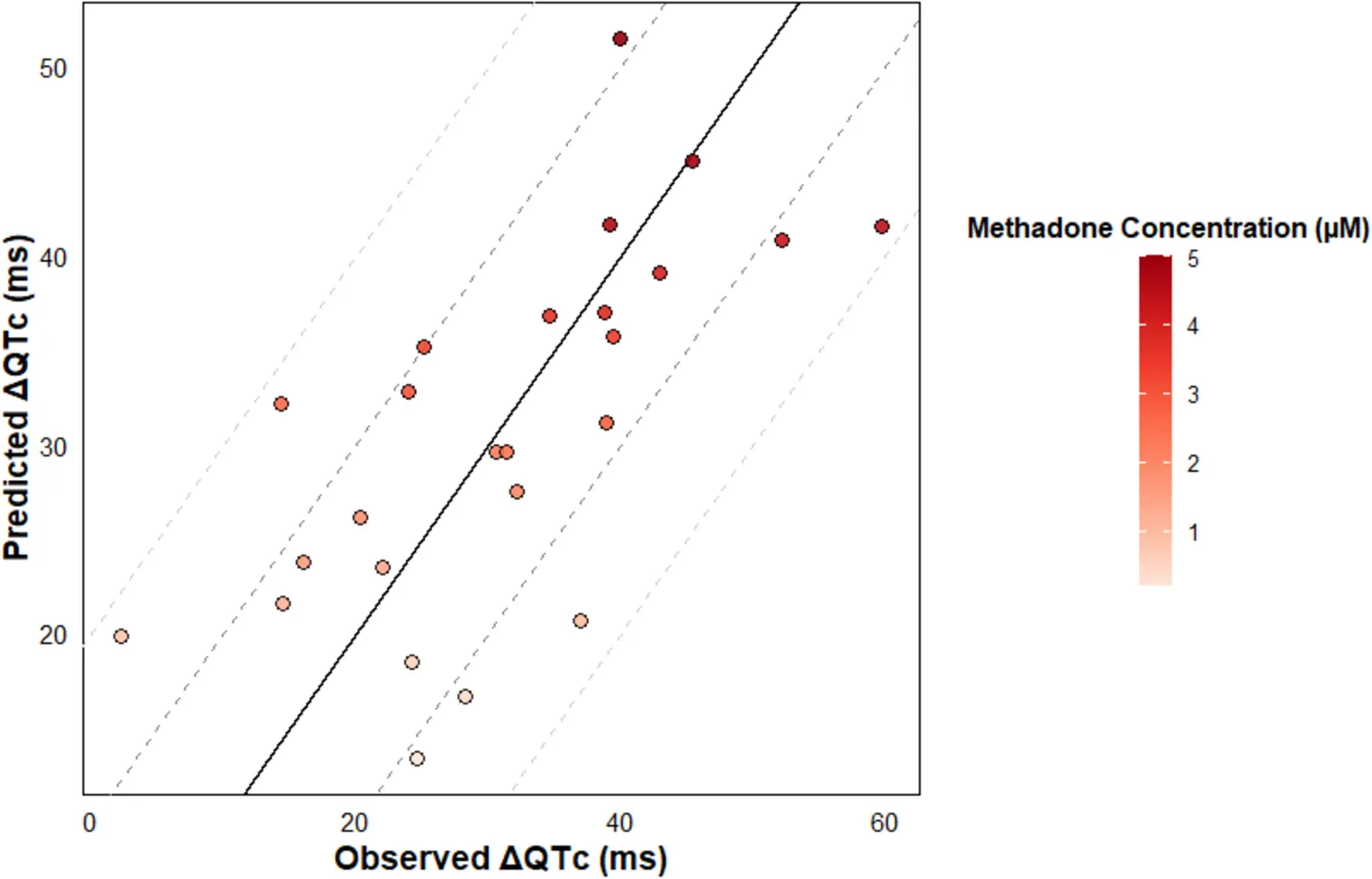
Goodness-of-fit for the predicted ΔQTc vs. observed ΔQTc intervals. The predicted ΔQTc values represent the within-subject difference between the full methadone simulation and a model without ion current inhibition. Observed ΔQTc was calculated as the observed QTc interval minus the mean QTc interval from the no-inhibition simulated model. The data were divided into 25 equally sized bins. The solid black line represents the line of identity. The dark grey dashed lines indicate a ± 10 ms deviation, and the light grey dashed lines represent a ± 20 ms deviation from this reference line. The methadone concentration for each group is represented by the color intensity of the dots, with darker dots corresponding to higher concentrations

The observed data yielded a slope of 8.83 and intercept of 409 ms, while the simulated data produced a slope of 9.28 and an intercept of 407 ms. Figure 3 displays the goodness of fit analysis by plotting the predicted ΔQTc values vs. the observed ΔQTc values.

**Fig. 4:**
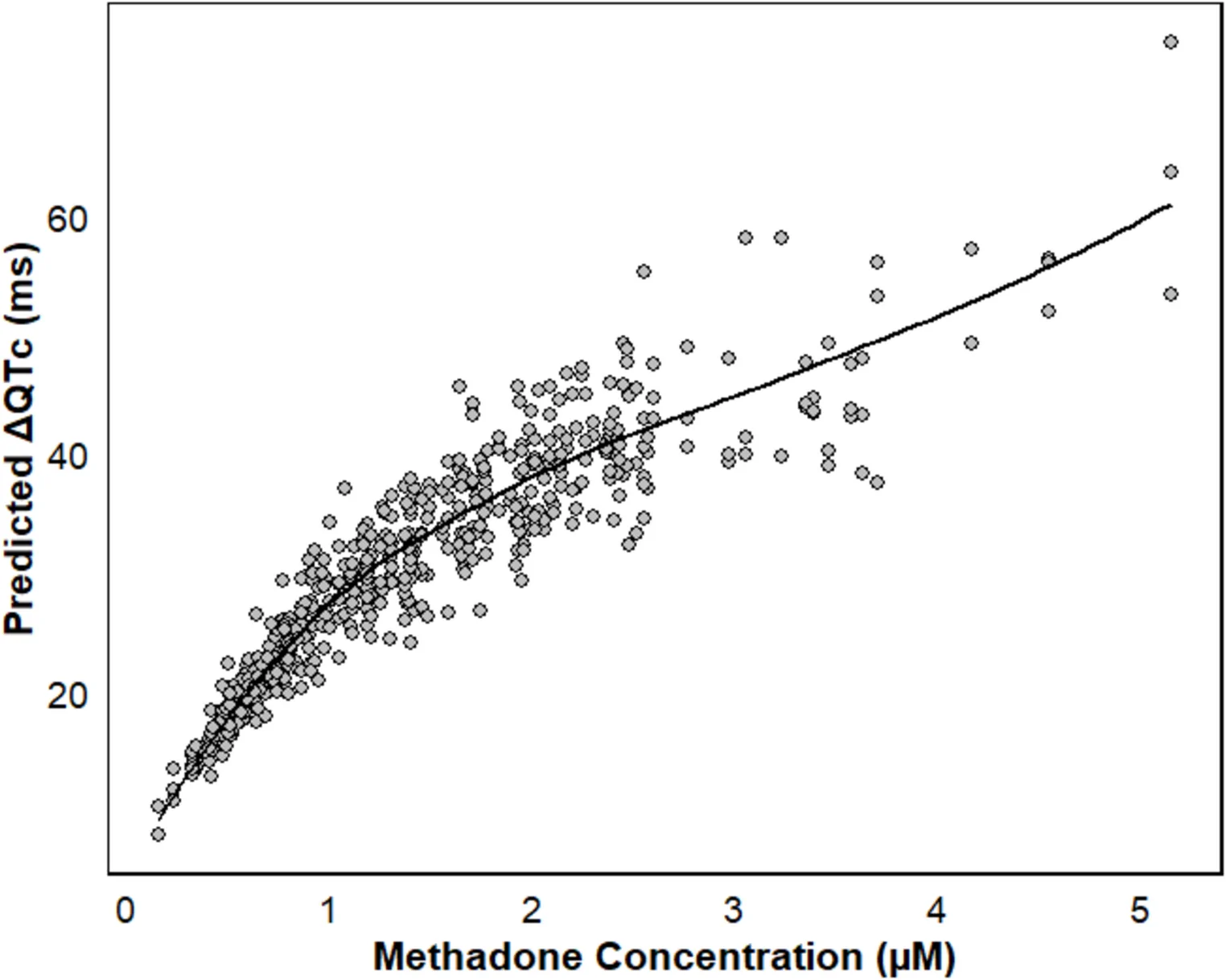
Predicted ΔQTc values plotted against methadone concentration. The predicted ΔQTc values represent the within-subject difference between the full methadone simulation and a model with no inhibition

The majority of comparisons (68%) exhibit a discrepancy of no more than 10 ms, with all differences falling within 20 ms. Figure 4 presents the predicted ΔQTc interval trend across the full range of methadone concentrations.

**Fig. 5:**
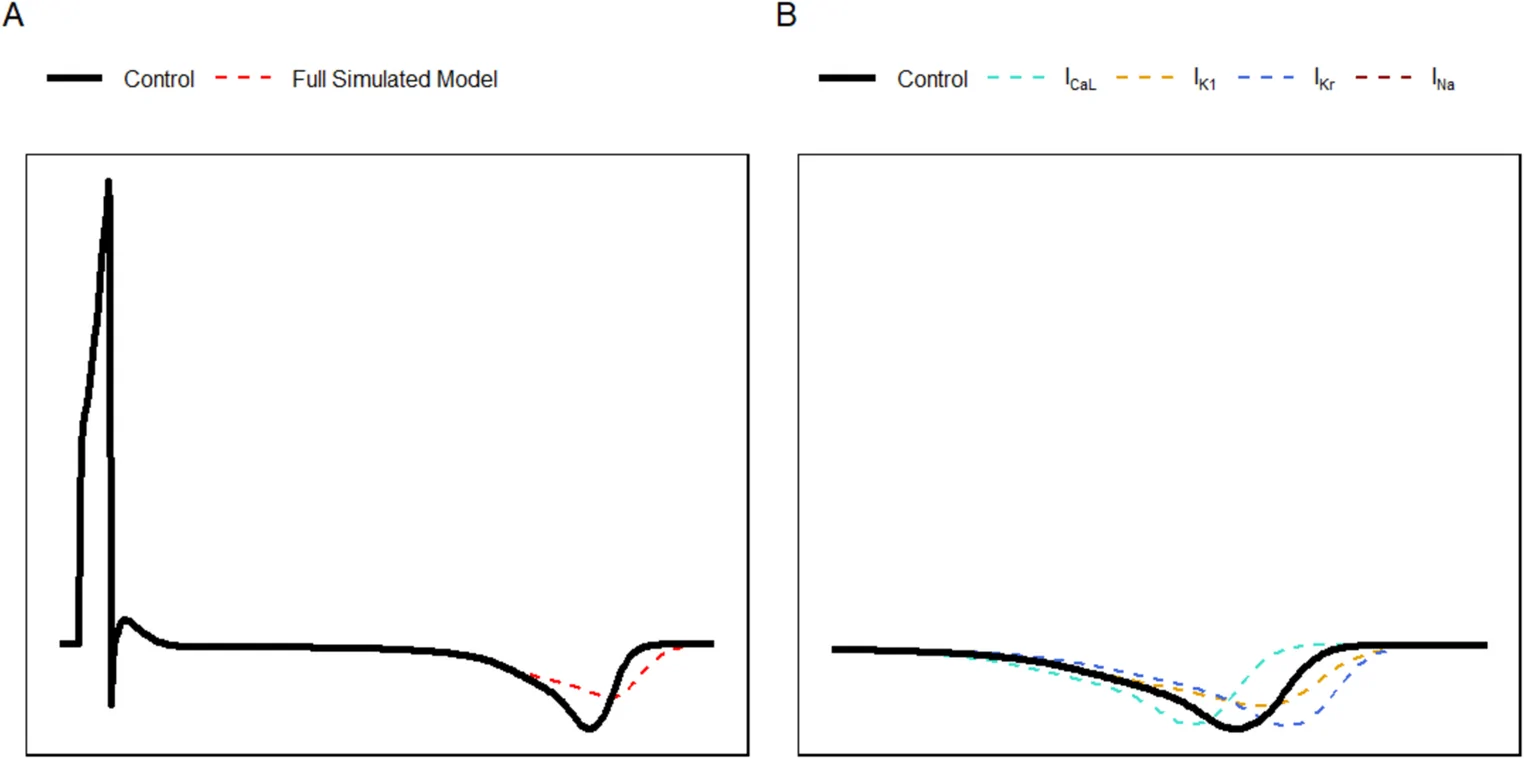
Representative pseudo-ECG from a virtual 41-year-old female at a methadone concentration of 1.28 µM. (**a**) The black trace represents the baseline pseudo-ECG without methadone-induced ion current inhibition, while the dashed red trace represents the ECG after simulated exposure to 1.28 µM methadone. This full model includes simultaneous inhibition of I_Kr_, I_K1_, I_Na_, and I_CaL_. (**b**) Isolated ion current contributions to QTc prolongation are shown. Each ion current (I_Kr_, I_K1_, I_Na_, and I_CaL_) was inhibited individually at methadone a concentration of 1.27 µM (33%, 47%, 1.6%, and 14% blockade, respectively) to assess its effect on QTc duration

Figure 5A illustrates the effect of methadone on the ECG of a virtual patient at the median observed concentration (1.28 µM). Methadone exposure increased the QTc interval from a baseline of 398 ms to 430 ms. Figure 5B demonstrates the effects of isolated ion current inhibition on the ECG of the same virtual patient.

The resulting QTc intervals (ms) were I_Kr_: 418, I_K1_: 422, I_CaL_: 358, and I_Na_: 399. These simulations suggest that I_Kr_ and I_K1_ inhibition were the primary drivers of methadone-induced QTc prolongation, and I_CaL_ inhibition shortened the interval, which is consistent with results from the action potential model (Fig. 1B).

Despite noticeable differences in T-wave morphology between the I_Kr_-only and I_K1_-only simulations, the peak of the T-wave was similarly displaced in both cases. Statistical comparisons showed no significant difference in QTc intervals between the individual I_Kr_ and I_K1_ inhibition conditions (*p* > 0.05), suggesting that methadone-induced inhibition of both I_Kr_ and I_K1_ have similar contributions to QTc interval prolongation. Each of the individual ion current inhibition models produced QTc intervals that differed significantly from both the full methadone simulation and the observed data (*p* < 0.05), highlighting the importance of incorporating the combination of ion current effects into the model.

To further quantify these contributions, mean ΔQTc from baseline was calculated at four concentration ranges (Table 5). Baseline QTc intervals were based on simulations without methadone-induced ion current inhibition. At moderate-to-high methadone concentrations, I_Kr_, I_K1_, and I_CaL_ played significant roles in modulating the QTc interval, causing changes approaching or exceeding 20 ms. In all four concentration ranges, I_K1_ inhibition alone resulted in greater QTc interval prolongation than I_Kr_ inhibition alone.

**Table 5:** Mean ΔQTc Intervals (ms) from Baseline Across Individual Ion Current Inhibition Models

Methadone Concentration	I_Kr_	I_K1_	I_CaL_	I_Na_
0-0.75 µM	10.1 ± 2.3	14.6 ± 2.3	-7.9 ± 2.2	0.03 ± 2.2
0.75–1.25 µM	15.5 ± 3.4	20.1 ± 3.4	-12.9 ± 3.1	0.1 ± 3.2
1.25-2 µM	20.5 ± 2.8	24.9 ± 2.8	-19.2 ± 2.6	0.1 ± 2.7
2-5.15 µM	27.5 ± 3.0	29.1 ± 2.9	-28.5 ± 2.6	0.2 ± 2.8

## Discussion

In this study, a computational electrophysiological model was used to fully replicate a clinical study of methadone and elucidate individual ion current contributions towards APD_90_ and QTc interval prolongation. The final model accurately predicted APD_90_ trends at therapeutic methadone concentrations and reproduced QTc intervals from chronic methadone users, with predictions falling within 1.5% of reported values across four concentration ranges. Additionally, the results demonstrate that I_Kr_ and I_K1_ contribute comparably to the prolongation of the AP and the QTc interval, whereas I_CaL_ plays a significant role in QTc interval shortening, particularly at high methadone concentrations. The findings highlight the complex interplay of methadone’s effects on cardiac ion currents and provide insight into how these interactions contribute to the observed clinical effect.

The current model reinforces that methadone-induced QT interval prolongation is primarily mediated through blockade of I_Kr_ and I_K1_, with notable attenuating effects from inhibition of I_CaL_ and I_Na_ [23, 25, 27–30, 32, 48]. Several studies have previously demonstrated the feasibility of using computational models to simulate both whole-study and patient-specific QTc values [49–51]. Additionally, Mikkelson et al. first simulated the relationship between methadone concentrations and QTc interval prolongation [23]. However, subsequent in vitro studies have expanded the information on methadone’s blockade of I_K1_ [25] as well as I_CaL_ [27–29] and I_Na_ [27, 29, 30]. These findings enable more comprehensive modeling of methadone’s electrophysiological impact. The present study aimed to quantify ion current contributions to the QTc interval across a wide range of observed methadone concentrations, rather than relying on a single overall mean. The findings align with those of Mikkelsen et al. [23]. while offering new mechanistic insights and underscoring the utility of computational modeling to improve the understanding of drug effects on cardiac electrophysiology.

Recent computational work by Zhang et al. [52] provided important mechanistic insight into methadone-associated arrhythmogenesis by demonstrating that blockade of the inward rectifier potassium current (I_K1_) can promote both early- and delayed-repolarization arrhythmias under specific electrophysiologic conditions. In contrast, the present study was designed to translate in vitro ion-channel inhibition data into clinically validated predictions of QTc interval prolongation, enabling direct comparison with observed concentration–QTc relationships in chronic methadone users while isolating the relative contributions of individual ion currents. Consistent with Zhang et al., the present study determined that I_K1_ inhibition contributes significantly to QT interval prolongation. Notably, the present study’s simulations demonstrate that methadone-induced QT interval lengthening is driven by both I_Kr_ and I_K1_ inhibition.

Klein et al. reported the effect of methadone on APD_90_ at three different concentrations [25]. The simulations in this study reproduced the experimental action potential trends at both therapeutic concentrations but did not capture the trend observed at the highest, supratherapeutic concentration. Notably, the results of the Klein et al. study were generated from swine left ventricular cardiomyocytes. Therefore, the differences in species-specific ion current densities, especially in I_K1_ and calcium handling, likely explain the divergent response [34]. At concentrations above 10 µM, methadone produces substantial block of I_CaL_, or other off‑target effects which in the swine preparation may dominate over K⁺ blockade to shorten APD. Because the human TNNP model weights I_Kr_/I_K1_ differently, I_CaL_ effects may not dominate to the same degree.

The use of a human left ventricular in silico model to predict APD_90_ trends in swine cardiomyocytes presents challenges, particularly due to differences in sensitivity to pharmacological effects on APD_90_ following I_Kr_ and I_K1_ blockade [34]. Additionally, differences in calcium handling could alter susceptibility to arrhythmogenicity, which may help explain the discrepancies observed between the simulated and observed action potentials at 10 µM [34]. Ultimately, the limited data and heterogeneity between the comparator study and the assumptions underlying the model constrain the ability to fully assess the validity of the APD_90_ predictions.

In cardiac risk assessment, in silico modeling can estimate in vivo electrophysiologic responses by simulating the effects of ion channel blockade on a one-dimensional strand of virtual cardiomyocytes. This modeling approach offers a systematic framework to understand arrhythmogenic potential by assuming that the net effect on ionic current results from the summative impact of the drug on each current individually [24]. Consistent with CiPA guidelines [19], which recommend evaluating IC_50_ values across several cardiac ion currents rather than focusing solely on I_Kr_, the current study incorporated methadone’s effects on I_Kr_, I_K1_, I_CaL_, and I_Na_. By integrating these features, the pseudo-ECG model allowed simulation of QT intervals across a virtual population and enabled validation against observed concentration–QTc relationships from chronic methadone users. This population-level framework demonstrated that incorporation of multiple ion current effects reproduced clinical QTc values within 1.5% across concentration strata. Furthermore, the pseudo-ECG simulations revealed that I_K1_ and I_Kr_ inhibition produced similar QTc prolongation despite differences in T-wave morphology, highlighting tissue-level phenomena that are not observable in isolated cardiomyocytes. By including all relevant ion currents the model was able to reproduce observed trends in both APD_90_ and QTc interval, demonstrating the value of comprehensive ion current assessment.

While the TQT study, as recommended by the FDA’s E14 guidelines [14], remains the standard approach for assessing a drug’s potential to prolong the QT interval, it is both time- and resource-intensive. In this context, the current study highlights the value of computational modeling as a complementary approach that can provide early-stage insight into proarrhythmic risk. By integrating in vitro data across multiple ion currents, the model was able to simulate human QTc interval responses to methadone and validate these predictions against clinical ECG data. These findings support the utility of in silico modeling, and particularly the CSS, as a cost-effective strategy for guiding cardiac safety assessments during drug development [18]. While QTc interval prolongation increases the risk for TdP, drug-induced TdP is most common with additional risk factors including overdose, drug interactions, and renal impairment, etc. The TNNP model adequately predicted QTc intervals at higher methadone concentrations, clinical data were not available for validation beyond 10 µM.

Given the limitations of in silico modeling several considerations should be made when interpreting the results of this study. First, the simulations and observed data were based on the racemic mixture, R,S methadone. Evaluation of ion current inhibition by the individual enantiomers was not possible due to missing IC_50_ values and a lack of validating data. Secondly, the TNNP model demonstrated markedly superior agreement with observed QT intervals relative to the other candidate models during the model selection procedure. However, the TNNP model does not include I_NaL_. Models of methadone that explicitly include I_NaL_ blockade result in minimal additional APD shortening once I_CaL_ blockade is incorporated [52]. This effect appears to be primarily driven by the relatively higher IC₅₀ values for methadone late sodium current compared with other ion currents, though some degree of model dependence cannot be excluded. Taken together, the low likelihood of I_NaL_ blockade affecting the study’s conclusions, combined with the preference to maintain consistency with previous methods, supported the use of the ten Tusscher model. Nonetheless, the absence of I_NaL_ may contribute to the model’s behavior at higher concentrations and we cannot evaluate contributions from currents that were not included in this study. The TNNP as other models does not capture full organ-level electrophysiology, and the results may be sensitive to heterogeneity assumptions. Absolute QT values and ventricular repolarization are influenced by the assumed transmural electrophysiological heterogeneity. In this study, we used the established TNNP-based transmural configuration implemented in Cardiac Safety Simulator, which has been extensively used and validated for simulation of drug-induced QT prolongation and cardiac safety assessment [18, 24, 49, 50]. Therefore, QTc predictions should be interpreted as model-derived surrogates of repolarization dynamics, not direct reconstructions of clinical ECGs. Additionally, the CSS employs physiological parameters representative of a Northern European population. Consequently, extrapolating the findings to other populations should be approached with caution. Finally, methadone’s effects were not evaluated at the individual level, and instead, the model outputs were compared based on grouped responses. The absence of subject-level demographic information, such as age and sex, prevented assessment of individual variability in QTc response to methadone.

## Conclusions

This study leveraged in silico human cardiomyocyte modeling to quantify the contributions of specific cardiac ion currents towards methadone-induced QTc prolongation. These findings offer a comprehensive perspective on the interplay between cardiac ion currents in this group of patients. In silico modeling was successfully used to extrapolate in vitro cardiac ion current effects to in vivo ECG outcomes. By validating model predictions against patient-level ECG data, the present work demonstrates that I_K1_ inhibition produces QTc prolongation comparable in magnitude to I_Kr_ blockade across therapeutic and supratherapeutic methadone concentrations. The identification of I_K1_ as a key underlying mechanism for methadone cardiotoxicity further supports its inclusion in translational in silico cardiac risk assessment.

## Data Availability

No datasets were generated or analysed during the current study.
